# Investigating the Association Between Salivary Profiles, Production-Related Variables, and Daily Lying Time in Dairy Cows: A Pilot Study

**DOI:** 10.3390/ani16162621

**Published:** 2026-08-21

**Authors:** Greta Veronica Berteselli, Emanuela Dalla Costa, María Dolores Contreras-Aguilar, Joel Fernando Soares Filipe, Marina López-Arjona, Gaia Pesenti Rossi, José Joaquín Cerón, Fernando Tecles, Elisabetta Canali

**Affiliations:** 1 Department of Veterinary Medicine and Animal Science, University of Milan, 26900 Lodi, Italy; emanuela.dallacosta@unimi.it (E.D.C.); joel.soares@unimi.it (J.F.S.F.); gaia.pesenti@unimi.it (G.P.R.); elisabetta.canali@unimi.it (E.C.); 2 Salilab-UMU, Interdisciplinary Laboratory of Clinical Analysis, Veterinary School, Regional Campus of International Excellence ‘Campus Mare Nostrum’, University of Murcia, Campus de Espinardo, 30100 Murcia, Spain; mariadolores.contreras@hotmail.com (M.D.C.-A.); jjceron@um.es (J.J.C.); ftecles@um.es (F.T.); 3 Department of Animal and Food Science, Universitat Autònoma de Barcelona, Cerdanyola del Vallès, Bellaterra, 08193 Barcelona, Spain; marina.lopez10@um.es

**Keywords:** animal welfare, accelerometer sensors, biomarkers, dairy cows, lying time, saliva, sialochemistry

## Abstract

Lying behaviour reflects cattle welfare response. This study aimed to investigate changes in salivary profiles and production variables based on lying time in dairy cows to identify potential biomarkers of rest-related changes. Over a three-month period, lying time was monitored in 15 healthy commercial dairy cows using accelerometric collars. Saliva samples were analyzed for relevant biomarkers. The LMM showed that salivary cortisol (β = 0.481, 95% CI: 0.204–0.758, *p* = 0.001) and total adenosine deaminase (tADA, β = 0.011, 95% CI: 0.005–0.017, *p* < 0.001) were positively associated with daily lying time, whereas creatinine kinase (CK) was negatively associated with daily lying time (β = −0.015, 95% CI: −0.030 to −0.001, *p* = 0.035). Metabolic stress can drive cows to prioritize lying, revealing a coping response to sub-optimal welfare. Increased salivary tADA reflects inflammatory conditions that alter animal behaviour by enhancing lying time. Increasing CK indicates muscle tissue injury, and physical strain may be due to acute restlessness or sustained standing. The findings suggest that changes in lying behaviour may be associated with changes in salivary analytes. The results are encouraging but require validation in larger and more diverse populations before routine application in dairy welfare assessment.

## Simple Summary

Monitoring how long dairy cows spend lying down is a common, non-invasive way to assess their welfare and comfort. This study investigated whether we could identify specific physiological “biomarkers” in saliva and production indicators that correlate with these resting patterns. Over a three-month period, 15 healthy dairy cows on a commercial farm were monitored. Precision livestock farming (PLF) technology, specifically accelerometer collars, was used to precisely track daily lying time. Saliva samples were collected at regular intervals to analyze various parameters. Our analysis revealed that specific markers, such as salivary cortisol, adenosine deaminase and creatine kinase, were associated with the resting behaviour of cows. These findings suggest that combining behavioural data from precision technology with physiological analysis can represent a promising, complementary tool to better understand cow welfare and improve herd management practices.

## 1. Introduction

The growing concern for dairy cows’ welfare in confinement housing has highlighted the need for a more thorough examination of cattle behaviour on farms to better understand the effect of the free-stall environment [1,2,3]. The free-stall barn is currently the predominant housing system due to its versatility and adaptability [4,5]. However, parlour facilities limit cows’ control over milking time, as this depends on management practices and parlour design [6]. Under normal conditions, dairy cows need 10 to 14 h of lying time and 3 to 5 h of feeding per day. The duration of rumination per day ranges from 7 to 10 h, while the remaining time is divided into activities such as eating, drinking, walking, standing and social interaction [7]. Nevertheless, natural behaviour expression by dairy cows in intensive farms is limited by environmental aspects (e.g., stall design, feed bunk design, flooring type) and management routines (e.g., milking time, grouping strategy, stock density, etc.) [1,8].

The behavioural time budget of cows can be assessed using continuous observations from precision livestock farming (PLF) technology (e.g., accelerometers) [9,10]. These systems overcome many limitations of traditional on-farm welfare assessments by enabling automatic and continuous recording of behaviours relevant to animal welfare and health in a time-efficient manner [11,12,13,14]. Scientific evidence suggests that cows affected by health problems such as mastitis, metritis, laminitis or metabolic disorders show altered behavioural patterns and time budgets, including changes in feed and water intake, feeding, rumination, and lying behaviour [15,16,17,18,19,20]. In particular, lying time, lying bout frequency and duration are recognized as useful indicators for dairy cow health and welfare status [21,22,23]. Lying behaviour assessment also provides valuable information on how cows interact with their environment [24,25,26,27,28]. The interaction with the environment includes not only changes in behavioural patterns but also induces physiological responses, including the release of chemical and endocrine mediators that trigger immune and inflammatory responses [1,29,30]. These signals act on the brain by triggering specific biochemical mechanisms and modify animal behaviour (e.g., apathy, fever, loss of appetite and thirst, reduction in social interaction and general activity) [31]. Due to contributions from both local (salivary glands) and systemic (blood) sources, saliva is an essential matrix for the search of disease biomarkers and assessing physiological status. Its primary advantages over blood include easy accessibility, non-invasive collection, and minimal stress for the animal [32,33,34].

This pilot study aimed to investigate possible changes in salivary profiles and animal production-related variables (somatic cell count (SCC); daily milk production (DPM); number of lactations) based on lying time in dairy cows to identify potential biomarkers of rest-related physiological changes.

The panel of salivary analytes included stress-related analytes (cortisol (sCo), total esterase (TEA), salivary alpha-amylase (sAA), and lipase (LIP)), immunity-related analytes (total adenosine deaminase (tADA) and isoenzymes (ADA1 and ADA2)) and tissue damage (creatine kinase (CK), total protein (TP)).

## 2. Materials and Methods

This pilot study was conducted as part of a broader project aimed at co-designing, developing, and validating a software platform powered by an algorithm integrating PLF data to provide information on animal welfare, as well as environmental and economic sustainability metrics. The objective of this platform was to support decision-making for stakeholders and consumers across the pig and dairy cattle value chains (Horizon 2020—ClearFarm-Co-designed Welfare Monitoring Platform for Pig and Dairy Cattle).

### 2.1. Animals and Housing

Fifteen high-producing, healthy Holstein–Friesian dairy cows, aged between 3 and 9 years (mean age: 4 ± 1.49), of different parity orders (mean parity: 2.26 ± 1.33, min = 1; max = 6), and at different lactation stages (stage 1: late dry period—15 days before delivery to first month of lactation; stage 2—30–90 days of lactation; stage 3—>90 days of lactation) were recruited. The cows involved in this study were part of the sample of animals considered for a larger study (Horizon 2020—ClearFarm-Co-designed Welfare Monitoring Platform for Pig and Dairy Cattle). Due to economic, personnel, and budgetary factors, the number of animals subjected to saliva sampling was limited.

This pilot study was conducted on a commercial intensive free-stall dairy farm located in the Lombardy region (North of Italy).

The farm utilized temperature, humidity, and cooling control systems, which were activated as necessary to prevent adverse conditions affecting the cows’ welfare and health. The environmental conditions were kept under control and were the same for all animals. Additionally, the animal density was calculated according to the legislation in force for animal welfare.

The housing and management are summarized in Table 1.

### 2.2. Animal Data Collection

The lying time of 15 cows was monitored with collars fitted with accelerometers (Connecterra B.V., Amsterdam, The Netherlands) over a three-month period (from February 2021 to May 2021). For the purpose of the present study, sensor data from a three-day window were considered, namely the lying time budget of the day of saliva collection, the previous day, and the subsequent day. The sensors recorded the behavioural time budget in seconds, continuously over 24 h.

The production parameters were extrapolated from the farm’s management system. Health status was assessed using farm veterinary records and physical examinations.

### 2.3. Saliva Sampling and Analytes

Saliva samples were collected from each animal on days 1 (T0), 45 (T1) and 90 (T2) of the trial. Sampling was conducted between 8:30 a.m. and 11:30 a.m., after the milking routine. Sampling was performed by introducing a commercial propylene sponge (La Griega E. Koronis, Madrid, Spain), cut into pieces of approximately 5 × 2 × 2 cm, clipped to a flexible thin metal rod, into the cow’s mouth under the tongue for 15–30 s. The sponge was then placed in a Salivette collection tube (Sarstedt, Aktiengesellschaft & Co., Nümbrecht, Germany). The samples were stored no more than four hours in ice until processing; the saliva was centrifuged at 3000× *g* for 10 min at 4 °C. The obtained saliva samples were stored for less than five months at −80 °C until analysis [35,36].

The salivary analytes evaluated in the sialochemistry profile were as follows: stress biomarkers such as sCo, TEA, sAA and LIP; immunity biomarkers such as tADA, ADA1, and ADA2; and tissue damage and metabolism biomarkers such as CK and TP. The analysis performed for each analyte and their functions are summarized in Table 2. The methods were validated for bovine saliva [35,36,37].

### 2.4. Data Analysis and Statistics

Statistical analyses were performed using SPSS Statistics Version 29.0 (IBM Corp., Armonk, NY, USA). The normality of daily lying time at each time point (T0, T1, and T2) was assessed using the Shapiro–Wilk test and descriptive statistics.

Potential multicollinearity among the fixed effects entered into the model was checked by estimating the Variance Inflation Factor (VIF) values; a VIF threshold of less than 3 was considered as the criterion for the absence of collinearity. VIF values ranged from 1.202 to 2.958, with the highest value observed for α-amylase (VIF = 2.958). Daily lying time was analyzed using a linear mixed model (LMM) with a normal probability distribution and identity link function. Cow identity was included as a random intercept to account for the repeated measurements obtained from the same animals. Assessment time (T0, T1 and T2) was specified as the repeated measure, and an autoregressive first-order covariance structure (AR1) was used to account for the correlation between repeated observations within cows. Fixed effects included α-amylase (sAA), lipase (LIP), total adenosine deaminase (tADA), creatine kinase (CK), total protein (TP), total esterase activity (TEA), salivary cortisol, somatic cell count (SCC), daily milk production (DMP) and number of lactations. Because the model used a normal distribution with an identity link function, the regression coefficients and their 95% confidence intervals are reported on the original response scale and have not been back-transformed. Statistical significance was defined as *p* < 0.05.

## 3. Results

The results of the different salivary biomarkers, lying behaviour, and production parameters measured in the populations are shown in Table 3.

The LMM identified significant associations between daily lying time and three salivary biomarkers. Salivary cortisol was positively associated with daily lying time (β = 0.481, 95% CI: 0.204–0.758, *p* = 0.001), as was tADA (β = 0.011, 95% CI: 0.005–0.017, *p* < 0.001). In contrast, CK was negatively associated with daily lying time (β = −0.015, 95% CI: −0.030 to −0.001, *p* = 0.035). No significant associations were observed for α-amylase, lipase, total protein, TEA, SCC, daily milk production, or the number of lactations (all *p* > 0.05). The random effect of cow identity accounted for a significant proportion of the residual variance (variance component = 197.136, Z = 2.145, *p* = 0.032), indicating substantial between-animal variability in baseline lying behaviour (Table 4 and Table 5).

## 4. Discussion

Saliva is considered a reliable matrix for the measurement of analytes associated with stress, metabolism, inflammation, and the immune system, while being less painful and invasive compared to blood sampling [35,37]. In the current study, a sialochemical profile was explored in dairy cows to identify potential biomarkers of rest-related changes. As evidenced in the literature, in commercial free-stall barns, dairy cows spend 10 to 14 h per day lying down, underlining the strong biological need of cows to rest [7]. Lying time is relevant not only for the adequate provision of rest and sleep, but it is also liked to rumination and rumen health, as lying cows are more prone to ruminate and produce saliva than standing ones, therefore reducing the risk of ruminal acidosis [38]. Moreover, the circulation through the udder is increased in lying cows, promoting udder function and milk production [25,39,40]. Therefore, adequate lying time is often considered a key aspect for dairy cow welfare [3,7,23,41,42,43].

The results of this study showed an association between the decrease in lying time and the increase in salivary CK concentration. Creatine kinase is an intracellular enzyme present in high concentrations in energy-intensive tissues, especially skeletal muscle, where it plays a significant role in energy homeostasis [44,45,46]. An increase in CK activity was observed following muscle injury or heightened physical exertion triggered by restlessness or by longer periods of standing [47]. Indeed, when a cow spends more time standing at the expense of normal rest times, the pressure inside the claw capsule increases and can produce hypoxia and ischemia, promoting the inflammatory process and consequently increasing the risk of lameness [3,39,48]. A stress and fatigue condition in cows with decreased lying time may be indicated by CK [44,45,46].

Cortisol is widely utilized as a key physiological biomarker for assessing acute stress responses in animals, through the activation of the hypothalamic–pituitary–adrenal (HPA) axis. Activation of the HPA axis triggers significant physiological shifts during acute stress, prioritizing adaptive responses at the expense of non-essential processes [4]. Consequently, nonadaptive functions such as growth, reproduction, and feeding are downregulated to conserve energy for immediate survival [49,50,51].

In intensive farming, discomfort or stress may arise from environmental and management factors (e.g., negative housing, stocking density, inadequate facilities, social-related problems) or clinical conditions (e.g., mastitis, ketosis, metritis) [1,3,5,52].

Additionally, in dairy cows, high milk yield can lead to a physiological burden, causing metabolic stress and triggering activation of the HPA axis with an increase in cortisol production [53,54]. The milk production per day of the animal sample resulted quite high (mean 37.9 ± 8.19 kg/day). These factors can affect cows’ lying behaviour [28,48,55,56], and under this conditions, dairy cows have been shown to prioritize lying over other behaviours (e.g., feeding, ruminating, social interactions) [4,24,42,57,58]. This may explain the significant positive association between salivary cortisol and lying time observed in this study. Moreover, the study found that the effect size for cortisol was greater than for other analytes, suggesting that cortisol changes could be more reliable than those of other analytes.

In dairy cattle, an increase in lying time coupled with elevated cortisol profiles can be indicative of negative welfare; it might indicate underlying pathological or environmental issues [4,23,57,58]. Alternatively, this could reflect a compensatory rest rebound following periods of stress. Therefore, the longer lying times observed in cows with higher cortisol levels should be interpreted as a coping mechanism under sub-optimal welfare conditions [59,60].

A similar mechanism can also explain the positive association found in this study between salivary tADA activity and lying time. tADA is an enzyme present in most tissues, but particularly in lymphoid tissues, where it is involved in the differentiation and development of immunological cells. Therefore, it is considered as a marker of cell-mediated immunity [61]. Specifically in cows, salivary tADA can be considered as a marker of inflammation and immunity, increasing after calving [62] or in animals with metritis [63]. Thus, an inflammatory condition affecting the animals’ the behavioural pattern and increasing lying time would also increase salivary tADA levels.

Despite all the animals being housed in the same conditions (i.e., environment, diet, and management), not all of them can exhibit the same physiological and behavioural response to the same stimulus. Therefore, the biomarkers’ outcomes should be interpreted considering the possible variations in individual plasticity, prior experience, and physiological condition [64,65]. Indeed, it was observed that during the peripartum period, cows experience systemic immune dysregulation and metabolic stress that can influence the levels of inflammatory biomarkers such as tADA and cortisol [36,66]. Finally, the changes observed in CK and tADA should be taken with caution due to a low size effect. For these reasons, the biomarkers significantly related to lying should be considered as promising complementary welfare indicators.

The primary limitation of this study was the small sample size. Future research should include a larger sample size and multiple farms to validate these findings and investigate potential variations in sialochemical profiles related to behavioural changes.

In addition, the observational design of the study precludes definitive conclusions regarding causality; thus, whether increased cortisol levels promote lying behaviour, lying itself causes spikes in cortisol, or both are influenced by an unaccounted-for confounding variable remains to be elucidated.

Moreover, ideally, the groups should have been more homogeneous in terms of lactation period. Due to the limited number of animals, this factor was not considered as a covariate.

As reviewed by Tucker and colleagues (2021) [23], the effects of parity and stage of lactation on lying behaviour remain inconsistent across studies. While some studies have reported longer lying times in multiparous cows, particularly during early lactation, others found no effect or even shorter lying times, suggesting that parity interacts with the stage of lactation and housing conditions rather than exerting an independent effect [21,23,67,68]. Likewise, lying behaviour changes throughout lactation, although these variations may reflect physiological adaptations associated with the transition period rather than changes in animal welfare [23,69,70]. Therefore, parity and stage of lactation should be considered as potential confounding factors when interpreting lying behaviour and should be included in future studies investigating behavioural and physiological indicators of animal welfare.

In future studies, factors such as different farming systems, climatic conditions, stocking density, and lactation phases should be considered to better explore the potential of saliva biomarker analysis. Particular attention should also be given to the quality and quantity of cubicles as they can affect the welfare of cattle [71].

Moreover, saliva samples were collected at only three time points over a 90-day period. This limited sampling frequency may have missed acute stress responses occurring between assessments, highlighting the need for higher-density longitudinal sampling in future research to fully characterize temporal biomarker dynamics.

In addition, it should also be considered that saliva secretion can be affected by diet and rumen cycles, which in turn differ among individual animals [72].

Finally, animals with both normal and abnormal lying times due to different conditions (stress and pathologic conditions) should also be considered in future studies. As highlighted by Tucker et al. (2021) [23], lying behaviour is influenced by a complex interaction of animal-related, management, and environmental factors and, therefore, should not be interpreted as a stand-alone indicator of welfare. These kinds of studies are needed to fully understand the complex relationships between behavioural response, individuality, and possible biomarkers to identify animal profiles related to welfare status.

## 5. Conclusions

The results of this study showed the potential utility of the sialochemical profile as a promising complementary tool for welfare assessment. A significant link between decreased lying time and elevated salivary CK was found, suggesting an association between the alteration of lying behaviour, metabolic fatigue, and potential physical strain. Additionally, the positive association between observed cortisol and tADA patterns with lying time could point out the complex interplay between the metabolic demands of lactation, HPA axis activation, individual physiological plasticity and the prioritization of adaptive behaviours to maintain homeostasis in the case of cortisol, and the presence of inflammatory conditions in the case of tADA. According to the findings, the changes in CK and tADA should be considered with caution due to a low size effect. On the contrary, the greater size effect can possibly make cortisol a more reliable salivary biomarker. In light of what we have discussed, these biomarkers should be considered promising complementary welfare indicators in the future.

By integrating sialochemical analysis with automated behavioural monitoring and production data (i.e., milk yield), it is possible to gain a more nuanced understanding of how individual cows experience their environment, paving the way for more precise, welfare-oriented herd management strategies. This study should be considered a pilot study, and further research should be performed to validate the promising findings in wider and more varied populations before routine use in dairy cow welfare assessments.

## Figures and Tables

**Table 1:** Information on farm management and housing.

Farm Management and Housing	
Farming type	Intensive and conventional
Breed	Holstein Friesian
Average number of dairy cows	320
Average number of heifers	120
Average length of productive life (months) ^1^	51
Barn type	Free-stall
Bedding (for milking cows)	Straw and mattress
Milking system	Parlour
Access to pasture or outdoor area	Dry cows, Outdoor area (1 month)
Mortality rate (%) of dairy cows ^2^	2

^1^ Calculated as the average number of months from birth to slaughter for all milking cows on study farms during 2021. ^2^ Total involuntary culling rates calculated based on reason for removal.

**Table 2:** Sialochemistry analysis. sAA: salivary alpha-amylase; LIP: lipase; tADA: total adenosine deaminase; CK: creatine kinase; TP: total protein; TEA: total esterase activity; sCo: salivary cortisol.

Analyte	Biomarker Category	Analysis
sAA (IU/L)	Stress (acute). sAA reflects autonomous nervous system (ANS) activation	Commercial kits from Beckman (Beckman Coulter Inc., Fullerton, CA, USA) using the Olympus automated chemistry analyzer (Olympus Diagnostica GmbH AU 400, Beckman Coulter, Ennis, Ireland)
LIP (IU/L)	Stress (acute). LIP increases after acute stress due to its secretion and is regulated by the sympathetic nervous system (SNS).	Commercial kits from Beckman (Beckman Coulter Inc., Fullerton, CA, USA) using the Olympus automated chemistry analyzer (Olympus Diagnostica GmbH AU 400, Beckman Coulter, Ennis, Ireland)
tADA and isoenzymes (ADA1 and ADA2) (IU/L)	Immunity. Biomarker of cell-mediators immunity	Commercial Diazyme kit (ADA-D assay kit, Diazyme Laboratories, Poway, CA, USA) using the Olympus automated chemistry analyser (Olympus Diagnostica GmbH AU 600, Beckman Coulter, Ennis, Ireland)
CK (IU/L)	Damaged tissue. Biomarkers of general metabolism and muscle damage	Commercial kits from Beckman (Beckman Coulter Inc., Fullerton, CA, USA) using the Olympus automated chemistry analyzer (Olympus Diagnostica GmbH AU 400, Beckman Coulter, Ennis, Ireland)
TP (g/L)	General metabolism	Commercial kits from Beckman (Beckman Coulter Inc., Fullerton, CA, USA) using the Olympus automated chemistry analyzer (Olympus Diagnostica GmbH AU 400, Beckman Coulter, Ennis, Ireland)
TEA (IU/L)	Stress (acute). TEA is associated to the SNS activation.	Method based on the hydrolysis of the substrate 4-nitrophenyl acetate (Sigma-Aldrich Co., St. Louis, MO, USA) by salivary esterases and adapted to the Olympus auto-mated chemistry analyser (Olympus Diagnostica GmbH AU 600, Beckman Coulter, Ennis, Ireland)
sCo (μg/dL)	Stress (long/acute-term). Salivary cortisol reflects the levels of free plasma cortisol after the activation of the hypothalamic–pituitary–adrenal (HPA) axis	Chemiluminescent immunoassay system (Immulite 1000, Siemens Healthcare Diagnostic, Deerfield, IL, USA)

**Table 3:** Mean and median values of salivary analytes, lying time, and production variables. sAA: salivary alpha-amylase; LIP: lipase; tADA: total adenosine deaminase; CK: creatine kinase; TP: total protein; TEA: total esterase activity; sCo: salivary cortisol; SCC: somatic cell count; DMP: daily milk production.

Analyte/Variable	Mean ± SD	Median (25th–75th IQR)
sAA	3.27 ± 2.74	1.60 (1.60–2.00)
LIP	2.67 ± 1.20	1.74 (1.74–2.66)
tADA	7.14 ± 3.84	6.12 (3.43–8.97)
ADA1	5.90 ± 3.71	4.86 (1.99–7.29)
ADA2	1.24 ± 0.88	0.93 (0.35–1.83)
CK	4.16 ± 2.34	3.21 (1.81–4.61)
TP	187.08 ± 48.67	182.78 (133.80–210.85)
TEA	45.30 ± 13.18	44.75 (30.87–57.81)
sCo	0.17 ± 0.08	0.14 (0.01–0.25)
Lying time	9.68 ± 1.23	9.68 (8.88–10.44)
N. lactations	2.28 ± 0.98	2.00 (1.00–2.64)
SCC	481,435 ± 556,443	113,000 (54,000–459,217)
DMP	37.91 ± 8.19	38.80 (32.05–45.10)

**Table 4:** Tests of fixed effects from the linear mixed model assessing the association between daily lying time, salivary biomarkers, and animal production-related variables. sAA: salivary alpha-amylase; LIP: lipase; tADA: total adenosine deaminase; CK: creatine kinase; TP: total protein; TEA: total esterase activity; sCo: salivary cortisol; SCC: somatic cell count; DMP: daily milk production.

Source	F	df1	df2	Sig.
Corrected Model	5.418	9	28	<0.001
sAA	0.066	1	28	0.799
LIP	0.564	1	28	0.459
tADA	15.929	1	28	<0.001
CK	4.917	1	28	0.035
TP	3.237	1	28	0.083
TEA	0.322	1	28	0.574
sCo	12.656	1	28	0.001
SCC	0.260	1	28	0.614
N. lactations	0.311	1	28	0.581
DMP	2.646	1	28	0.115

**Table 5:** Parameter estimates and 95% confidence intervals from the linear mixed model for daily lying time. sAA: salivary alpha-amylase; LIP: lipase; tADA: total adenosine deaminase; CK: creatine kinase; TP: total protein; TEA: total esterase activity; sCo: salivary cortisol; SCC: somatic cell count; DMP: daily milk production.

Model Term	Coefficient (β)	Std. Error	95% CI Lower	95% CI Upper	Sig.
Intercept	99.12	14.832	68.737	129.503	<0.001
sAA	0.001	0.0043	−0.008	0.010	0.799
LIP	0.007	0.0092	−0.012	0.026	0.459
tADA	0.011	0.0028	0.005	0.017	<0.001
CK	−0.015	0.0070	−0.030	−0.001	0.035
TP	−0.001	0.0003	−0.001	7.234 × 10^−5^	0.083
TEA	0.001	0.0009	−0.001	0.002	0.574
sCo	0.481	0.1352	0.204	0.758	0.001
SCC	1.188 × 10^−6^	2.3314 × 10^−6^	−3.588 × 10^−6^	5.963 × 10^−6^	0.614
N. lactations	1.638	2.9367	−4.377	7.654	0.581
DMP	−0.026	0.0161	−0.059	0.007	0.115

Because the model used a normal distribution with an identity link function, the regression coefficients and their 95% confidence intervals are reported on the original response scale and have not been back-transformed.

## Data Availability

The data presented in this study are available on request from the corresponding author.
